# Histone acetyltransferase PCAF Up-regulated cell apoptosis in hepatocellular carcinoma via acetylating histone H4 and inactivating AKT signaling

**DOI:** 10.1186/1476-4598-12-96

**Published:** 2013-08-27

**Authors:** Xin Zheng, Xiaohong Gai, Feihu Ding, Zhongtang Lu, Kangsheng Tu, Yingmin Yao, Qingguang Liu

**Affiliations:** 1 Department of Hepatobiliary Surgery, the First Affiliated Hospital of Xi’an Jiaotong University, 277 Yanta West Road, Xi’an, Shaanxi 710061, China

**Keywords:** PCAF, Hepatocellular carcinoma, AKT signaling, Histone H4, Apoptosis

## Abstract

**Background:**

PCAF is an important intrinsic histone acetyltransferases. This study tried to establish the effect of PCAF on HCC cell apoptosis.

**Method:**

Both *in vitro* and *in vivo* experiments including IHC, DAPI staining, caspase 3/7 activity assay, BrdU assay, MTT assay, western immunoblotting and co-immunoprecipitation were used here.

**Results:**

PCAF was found to be expressed at the low level in most of HCC cell lines. PCAF overexpression induced cell apoptosis and growth arrest with increased Histone H4 acetylation and inactivation of AKT signaling in Huh7 and HepG2 cells. The opposite results were obtained by silencing PCAF in Hep3B cells. The co-immunoprecipitation assay confirmed that PCAF protein was bound with histone H4 protein in the nucleus of Hep3B cells. Finally, the *in vivo* experiment confirmed the findings mentioned-above.

**Conclusion:**

These data identified PCAF promotes cell apoptosis and functions as a HCC repressor through acetylating histone H4 and inactivating AKT signaling.

## Introduction

Hepatocellular carcinoma (HCC) is currently the fifth most common malignancy and the third most frequent cause of cancer death worldwide [1]. The statistical data show that HCC is the second most prevalent cause of cancer deaths for male and the third for female in China [2]. Moreover, the incidence of HCC in USA and west Europe countries keeps rising each year [3]. Due to lack of the distinct clinical manifestation and the aggressive feature of malignancy, most of HCC patients are diagnosed at the advanced stage and have no chance to receive the curative treatments like liver transplantation and radical liver resection, which results in the unfortunate prognosis. Therefore, it is urgent to figure out the pathogenesis of HCC and develop novel tumor markers and target therapies.

P300/CBP-associated factor (PCAF), a well-known histone acetyltransferases (HAT), was established in the research about the oncogenic function of adenoviral E1A which showed PCAF competed with E1A for binding to P300/CBP and in turn repressed cellular transformation [4]. The same investigation simultaneously demonstrated that PCAF had the intrinsic HAT activity which was found to be attributed to transcriptional activation [5]. Analysis of the sequence of PCAF protein reveals that the C-terminal half of PCAF contains the central HAT domain (within residues 725–819) which is highly homologous to the yeast GCN5 (yGCN5) [6]. Later, the functional experiment shows that the N-terminal structure of PCAF, which is different with yGCN5, is necessary for nucleosomal acetylation induced by the HAT domain of PCAF [4]. In our previous studies, PCAF was found to be frequently down-regulated in HCC tissues compared to adjacent liver tissues as assessed by immunohistochemistry (IHC) staining and down-regulation of PCAF in tumor specimens was negatively associated with promising survival after liver resection [7].

Among the various epigenetic regulatory mechanisms that cause alteration of gene expression, histone acetylation has been considered as one of most significance [8]. The amino terminus of histones extends from the nucleosomal core and could be modified by acetyltransferases or deacetylases. This modification leads to relaxation of chromatin structure facilitating transcriptional factors to bind with relevant promoters of target gene sequences and consequently controls numerous cell signal pathways {Shogren-Knaak, 2006 #14} [9]. Through regulating lots of cell pathways which control cell fate simultaneity, histone acetylation has been found to contribute to inhibit the growth and metastasis of gastrointestinal cancers including gastric cancer, colorectal cancer and HCC [10,11]. Yamashita et al. found that down-regulating acetylation of histone H4 by histone deacetylase inhibitor trichostatin A (TSA) resulted in cell-cycle arrest and apoptosis of HCC cells (HepG2 cells and Huh7 cells, respectively) [12]. The results from other groups also confirmed the pro-apoptotic effect of acetylation of histone H4 on HepG2 cells [13]. Interestingly, Lai and his colleagues found that acetylated histone H4 inactivated AKT signaling and consequently leaded to cell apoptosis in HCC [14]. Recently, there are more evidences confirming inhibition of AKT signaling is involved in increased cell apoptosis and growth arrest induced by acetylating histone H4 in several cancers including diffuse large B-cell lymphoma [15], non-small cell lung cancer and ovarian cancer [16]. AKT is a well-known serine/threonine kinase regulating its downstream effectors that affect critical cellular processes. It has been found that AKT signaling mediates cell apoptosis and growth via distinct ways such as inactivating cell cycle inhibitors (p27 and p21) [17], inhibiting pro-apoptotic genes (BAD and BIM), promoting cell cycle proteins (c-Myc, cyclin D1) [18] and degrading the tumor suppressor protein p53 [19].

Here, we tried to address the following questions: 1. Does PCAF affect cell apoptosis of HCC cells? 2. Are AKT signaling and histone H4 involved in the pro-apoptotic action of PCAF on HCC? 3. Does PCAF repress the growth of HCC xenografts?

## Materials and methods

### Materials

DMEM medium, RPMI 1640 medium, FBS and trypsin/EDTA were from Invitrogen Co. (Carlsbad, CA, USA). The PCAF expressing plasmid and its empty plasmid pCMV6-Entry were both purchased from Origene Technologies Inc. (Rockville, MD, USA). PCAF siRNA sequences were obtained from Santa Cruz Biotechnology (Catalog No.: sc-36198, Santa Cruz, CA, USA). The 18 s rRNA TaqMan probe (Hs99999901_s1) and PCAF TaqMan probe (Hs00187332_m1) were purchased from Applied Biosystems (Carlsbad, CA, USA). The rabbit monoclonal PCAF antibody, rabbit polyclonal phospho-AKT antibody (which can be used to detect phosphorylated AKT1, AKT2 and AKT3), AKT antibody (which can be used to detect total proteins of AKT1, AKT2 and AKT3), rabbit polyclonal acetyl-histone H4 (Lys16) antibody and rabbit polyclonal histone H4 antibody were from Cell Signaling (Danvers, MA, USA). The mouse monoclonal β-actin antibody and 4,6-diamidino-2-phenylindole (DAPI) were from Boster Biotechnology (Wuhan, China). The Caspase-Glo® 3/7 Assay kit and Apo-ONE® Homogeneous Caspase-3/7 Assay were from Promega (Madison, WI, USA). The terminal deoxynucleotidyl transferase-mediated dUTP-biotin nick end labeling assay (Tunel) kit (Catalog No.: KGA7025) was from KeyGEN BioTECH (Nanjing, China). The IHC detection kit (Catalog No.: SP-9001) was purchased from ZSGB Bio. (Beijing, China).

### Cell culture

HCC cell lines (Hep3B, HepG2, PLC/PRF5 and SK Hep1) were obtained from the American Type Culture Collection (Manassas, VA, USA) and Huh7 cell line was a kind gift from Prof. Kefeng Dou (Department of Hepatobiliary Surgery, Xijing Hospital, Fourth Military Medical University). SK Hep1, Hep3B cells and PLC/PRF5 were cultured in complete MEM medium with 10% FBS. Huh7 cells were grown in DMEM medium with 10% FBS. HepG2 cells were cultured in RPMI 1640 medium with 10% FBS.

### Establishment of PCAF stable transfectant clones

PCAF expressing plasmid was transfected into Huh7 cells using FuGENE6 Transfection Reagent from Promega (Madison, WI, USA) as PCAF-expressing Huh7 cells (Huh7 PCAF cells). The pCMV6-Entry plasmid was transfected into Huh7 cells as the control cells (Huh7 Control cells). Stable transfection for both Huh7 PCAF cells and Huh7 Control cells was obtained after 2-week selection with Geneticin (G418) from Invitrogen (Carlsbad, CA, USA) at a dose of 600 μg/mL.

### RNAi transfections

siRNA sequences against PCAF (Catalog No.: sc-36198) and the scramble siRNAs (Catalog No. sc-37007) were both from Santa Cruz Biotechnology (Santa Cruz, CA, USA). Hep3B cells were seeded at the concentration of 0.2 × 10^6^ per well in six-well plates and grown for overnight. Then tumor cells in each well were transfected with 100 nM siRNAs using Lipofectamine RNAi MAX Reagent (Invitrogen, CA, USA) according to the manufacturer’s instructions. The cells were used for further experiments at 48 h after transfection.

### Quantitative real-time reverse transcription polymerase chain reaction (qRT-PCR)

Total RNA was isolated from HCC cell lines using the Rneasy kit from Qiagen Co. (Valencia, CA, USA). cDNA synthesis was carried out using the High Capacity cDNA Reverse Transcription Kit from Applied Biosystems (Carlsbad, CA, USA) to transcribe 2 μg of total RNA. qRT-PCR was performed using ABI TaqMan Gene Expression assays in an ABI 7300 system. PCAF expressing plasmid was used to make the standard curve as the standard sample and 18 s rRNA was regarded as internal control. The mRNA level of PCAF was normalized to 18 s rRNA mRNA level in the same sample.

### Co-Immunoprecipitation assay and western immunoblotting

Co-immunoprecipitation assay was carried to examine the interaction between PCAF protein and histone H4 protein in Huh7 PCAF cells. Then, total protein lysate was obtained in immunoprecipitation buffer (50 mM Tris–HCl, pH 8.0, 150 mM NaCl, 5 mM EDTA, 0.5% NP-40, 2 μg/ml aprotinin, 1 μg/ml leupeptin, 1 mM PMSF, 1 mM sodium vanadate and 10 mM sodium fluoride). Next, the lysate was precleared with protein A/G-agarose beads. Total protein in supernatants was qualified by BCA method. Total protein was diluted into 1 μg/μL with PBS and mixed with primary antibodies against PCAF and histone H4 or IgG. The mixtures were shaken on rotating shaker at 4°C for overnight. The supernatant was collected and proceeded to immunoblotting assay.

Briefly, 30 μg protein samples were separated by denaturing gel electrophoresis. After transferred to PVDF membrane, blots were probed overnight with the primary antibodies respectively. After washed 3 times by TBST, blots were then incubated with the relevant secondary antibodies conjugated with HRP, and signals were visualized using the HyGLO HRP detection kit from Denville (Metuchen, NJ, USA) [20]. β-actin was measured as internal control.

### Cell proliferation and cell viability assays

For the proliferation assay, HCC cells were seeded into 96-well plates at 5000 cells per well for 24 hours and assessed using the BrdU ELISA kit from Roche Co. (Indianapolis, IN). The 3-(4, 5-dimethylthiazol-2-yl)-2,5-diphenyl tetrazolium bromide (MTT) assay was used to assess cell viability at 24, 48, 72 and 96 hours.

### Cell apoptosis detection

We performed three distinct assessments to get the convincing results of cell apoptosis here. First of all, cell apoptosis was measured by fluorescence microscopy to identify apoptotic nuclear changes (chromatin condensation and nuclear fragmentation) after staining cells with DAPI. Next, the Caspase 3/7 activity assay was conducted using the Apo-ONE® Homogeneous Caspase-3/7 Assay kit as described in our previous studies [21]. Finally, cell apoptosis was assessed by flow cytometry assay. Populations of apoptosis cells were determined by staining cells with annexin V–FITC and PI labeling, according to the manufacturer’s recommendations (Alexa Fluor® 488 annexin V/Dead Cell Apoptosis Kit, Invitrogen, CA, USA). FACS analysis was performed using a FACSCalibur (Becton Dickinson, San Jose, CA).

### *In vivo* experiments

Two million Huh7 PCAF cells or Huh7 Control cells suspended in 150 μL of Matrigel were inoculated subcutaneously into the flanks of 4 to 6 weeks old male nude mice. Tumor sizes were measured with calipers every 5 days. Mice were censored when the tumor volume reached 1000 mm^3^. All experimental protocols were approved by the institutional animal care and use committee of our hospital. The IHC staining assay was performed to detect the protein expression of PCAF, acetyl-histone H4 and phospho-AKT in the xenograft tissues. The cell apoptosis in the xenograft tissues was measured by TUNEL assay according to the manufacturer’s guidelines. The details of IHC protocal have been described previously [22].

### Statistical analysis

All experiments were performed in triplicates, repeated 2–3 times. And all data are expressed as means and standard errors of the mean. Differences between groups were compared with the Mann–Whitney test or Student-*t* test. A P value of < 0.05 was used for significance. All statistical analysis was performed using PRISM 4 (Graphypad, La Jolla, CA, USA).

## Results

### The PCAF expression in HCC cell lines

**Figure 1:**
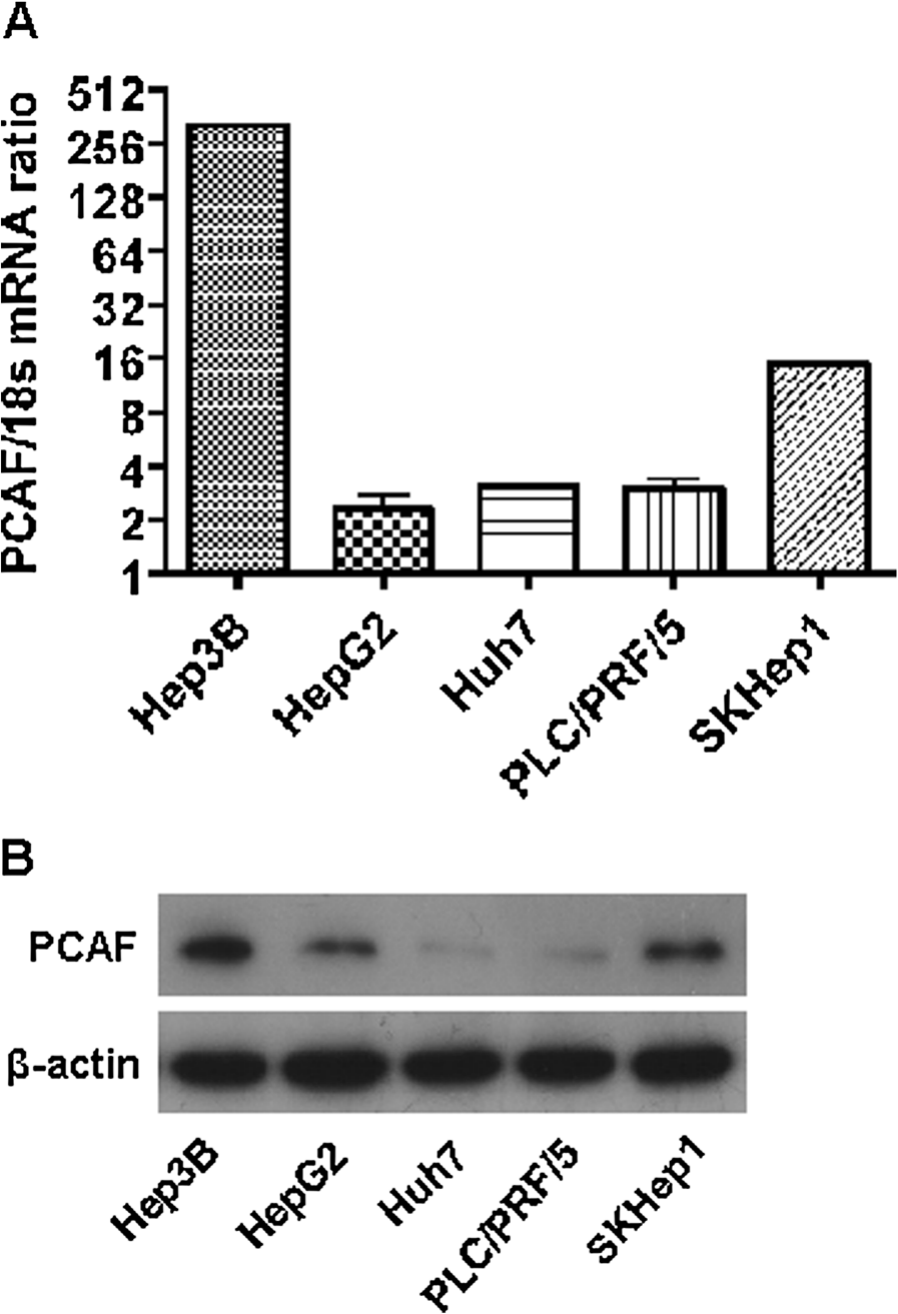
**The expression of PCAF in HCC cell lines. (A)** The mRNA of PCAF in 6 kinds of HCC cell lines was examined by qRT-PCR; **(B)** The protein expression of PCAF in 6 kinds of HCC cell lines was examined by Immunoblotting.

To investigate the level of PCAF in HCC cell lines and select the appropriate cell models for the further experiment, we detected the mRNA and protein expression of PCAF in Hep3B, HepG2, Huh7, PLC/PRF/5 and SKHep1 cells by qRT-PCR and immunoblotting. As shown in Figure 1A, Hep3B cell expressed the highest mRNA level of PCAF, while the mRNA expression of PCAF in Huh7, HepG2 and PLC/PRF/5 cells was relatively low. The results of immunoblotting assay verified these findings (Figure 1B), as well. Thereby, Huh7 cells were selected for PCAF overexpression experiment here, while Hep3B cells were used in PCAF knockdown experiment.

### Forced expression of PCAF induced cell apoptosis and growth arrest in HCC cells

**Figure 2:**
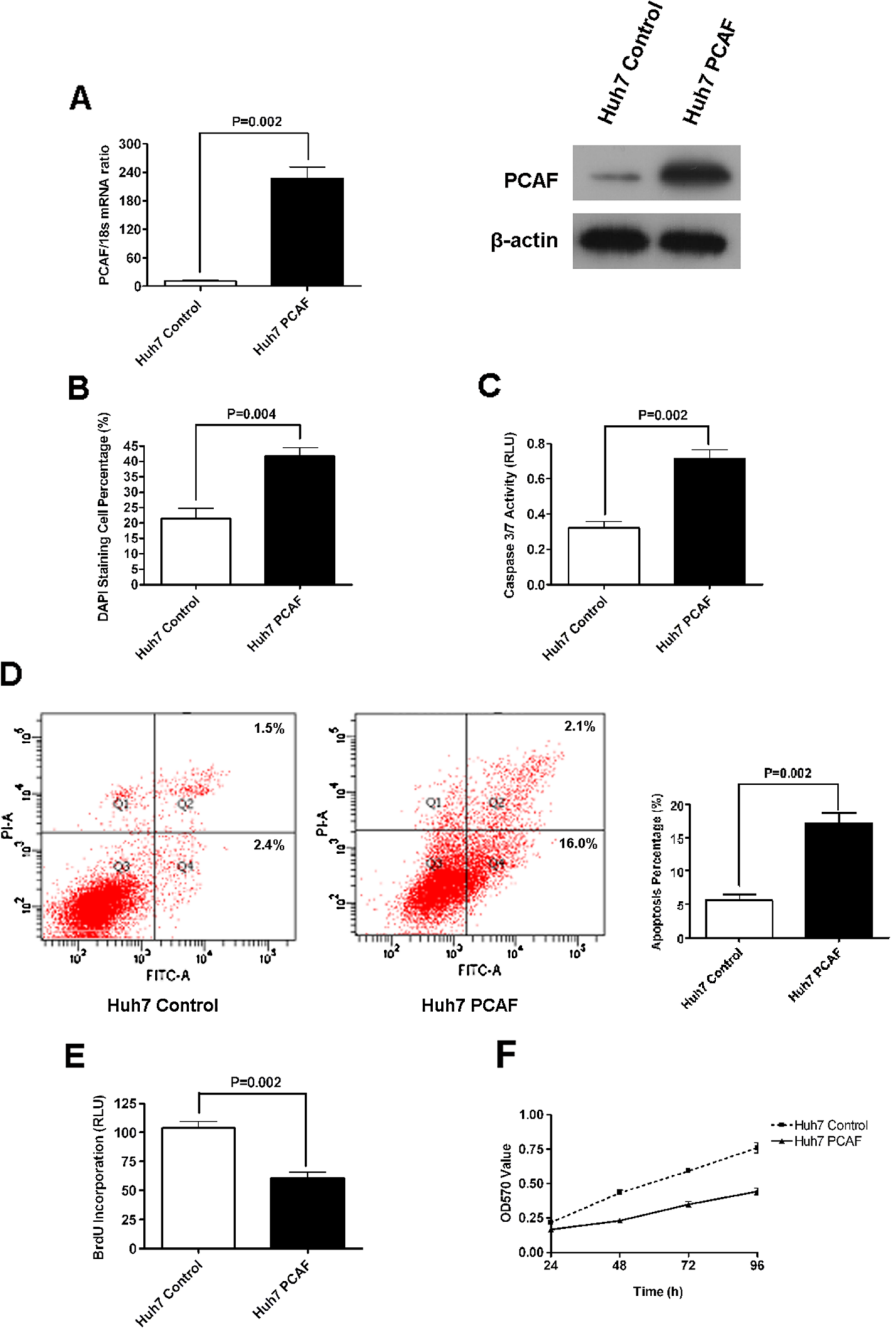
**Overexpression of PCAF induced cell apoptosis and repressed proliferation in Huh7 cells. (A)** At the levels of both mRNA and protein, PCAF expression is increased by PCAF expressing plasmid in Huh7 cells; **(B)** Forced expression of PCAF induced a significant increase in apoptosis of Huh7 cells as assessed by staining with DAPI followed by fluorescence microscopy (P < 0.004); **(C)** The activity of the pro-apoptotic caspase 3 and 7 also showed up-regulated after ectopic expression of PCAF (P = 0.002); **(D)** Flow cytometry assay showed that the percents of early apoptosis cells and late apoptosis cells was increased 2–3 folds in Huh7 PCAF cells than in Huh7 Control cells(P = 0.002); **(E)** Cell proliferation as measured by BrdU incorporation was inhibited by forced expression of PCAF(P = 0.002); **(F)** As assessed by MTT assays, forced expression of PCAF was found to reduce viability of Huh7 cells at all four time points significantly.

To determine the effect of PCAF on the growth of HCC cells, we established Huh7 clones which over-expressed PCAF stably by the PCAF expressing plasmid. As assessed by qRT-PCR and immunoblotting assay, the mRNA and protein expression of PCAF in Huh7 PCAF cells was significantly higher than in Huh7 Control cells (Figure 2A). The percentage of DAPI staining cells was around 40% in Huh7 PCAF cells, which was apparently higher than 20% in Huh7 Control cells (Figure 2B). Forced expression of PCAF was found to increase the caspase 3/7 activity by about 2 folds in Huh7 cells (Figure 2C). Flow cytometry apoptosis assays also showed that the percents of apoptosis cells including both early apoptosis cells and late apoptosis cells were increased 2–3 folds in Huh7 cells by PCAF overexpression, as shown in Figure 2D. Consistently, forced expression of PCAF suppressed cell proliferation of Huh7 cells. As assessed by luminometer, BrdU incorporation in Huh7 cells was decreased to about 50% after overexpression of PCAF (Figure 2E). The MTT experiments showed that forced expression of PCAF reduced viability of Huh7 cells at all four time points significantly, as shown in Figure 2F.

To verify the pro-apoptotic activity of PCAF on HCC cells, we increased the expression of PCAF in another kind of HCC cells with low level of PCAF - HepG2 cell, as shown in Additional file 1: Figure S1 A. The percentage of DAPI staining cells in HepG2 cells with high level of PCAF (HepG2 PCAF) was significantly higher than one in HepG2 cells with low level of PCAF (HepG2 Control) (Additional file 1: Figure S1 B). Consistently, the activity of caspase 3/7 of HepG2 cells was up-regulated after enforcing PCAF expression, as well (Additional file 1: Figure S1 C). These results demonstrated that PCAF promoted apoptosis of HepG2 cells *in vitro.*

### Knockdown of PCAF repressed cell apoptosis and accelerated proliferation in Hep3B cells

**Figure 3:**
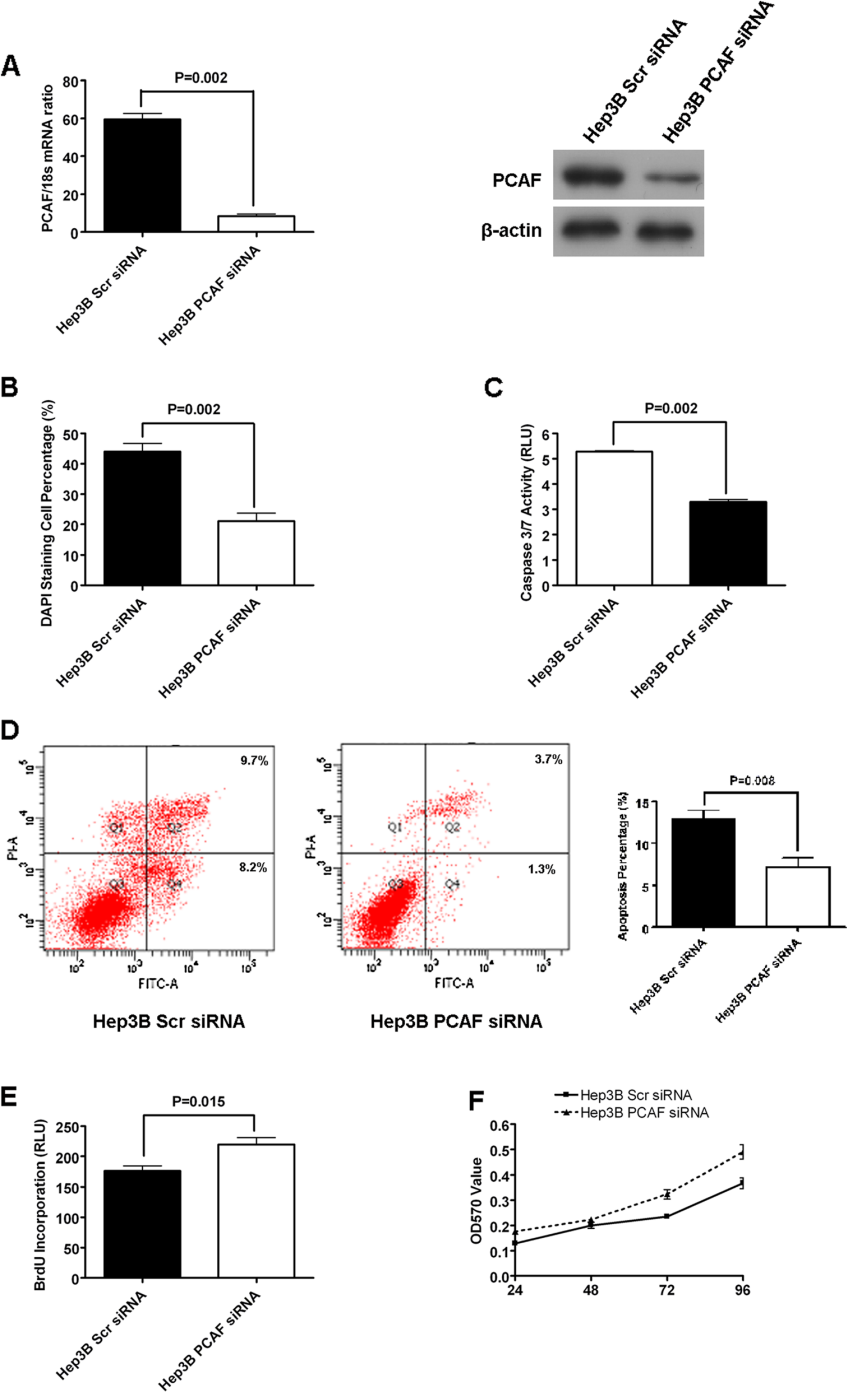
**Knockdown of PCAF suppressed cell apoptosis and promoted proliferation in Hep3B cells. (A)** The mRNA and protein expression of PCAF is down-regulated by siRNA structure against PCAF in Hep3B cells; **(B)** DAPI staining assay showed that knockdown of PCAF repressed cell apoptosis of Hep3B cells significantly (P = 0.002); **(C)** The activity of caspase 3/7 was decreased greatly by knockdown of PCAF in Hep3B cells (P = 0.002); **(D)** The percents of both early apoptotic cells and late apoptotic cells of Hep3B PCAF siRNA cells was decreased by around 50% as assessed by flow cytometry (P = 0.008); **(E)** Silencing PCAF was found to promote proliferation of Hep3B cells in BrdU incorporation ELISA assay apparently; **(F)** MTT assay displayed that knockdown of PCAF facilitated cell growth of Hep3B cells significantly.

To further determine the effect of PCAF on the cell apoptosis and proliferation of HCC cells, we silenced PCAF expression in Hep3B cells. The results of both qRT-PCR and immunoblotting assays showed that PCAF expression in Hep3B cells was decreased successfully by siRNAs transfection (Figure 3A). The percents of DAPI staining cells in Hep3B PCAF siRNA group was significantly lower than those in Hep3B Scr siRNA group (Figure 3B). Caspase 3/7 activity of Hep3B cells was decreased by about 40% after knockdown of PCAF (Figure 3C). Flow cytometry assay also showed that the percent of apoptosis cells including both early apoptosis cells and late apoptosis cells in Hep3B PCAF siRNA group was decreased by around 50% (Figure 3D). On the other hand, silencing PCAF was found to promote proliferation of Hep3B cells apparently by BrdU incorporation ELISA assay (Figure 3E). Correspondingly, the results of MTT assay displayed that knockdown of PCAF facilitated cell growth of Hep3B cells significantly, as well (Figure 3F).

### PCAF acetylated histone H4 directly and inhibited AKT signaling

**Figure 4:**
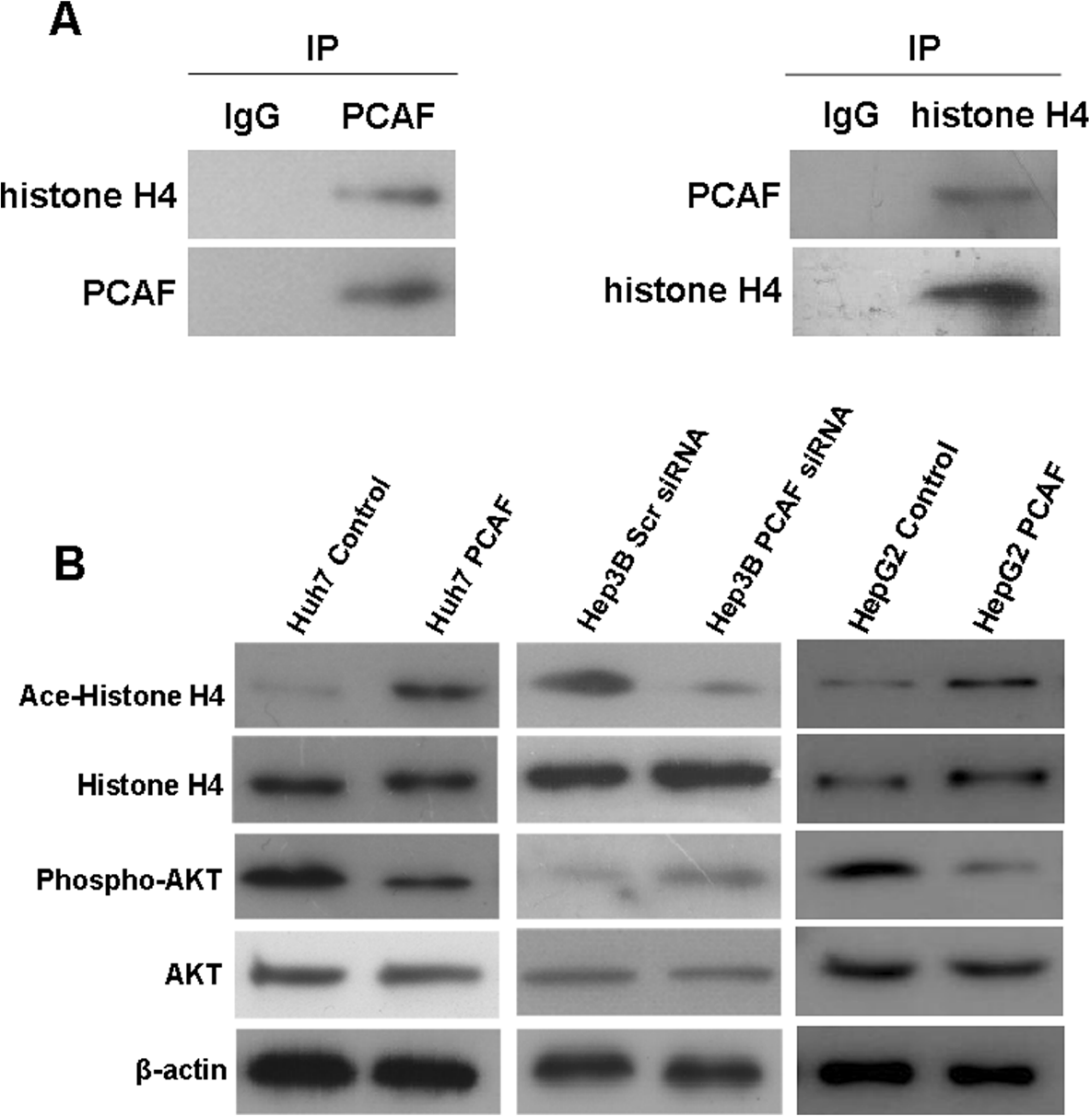
**PCAF regulated acetylation of histone H4 and phosphorylation of AKT in HCC. (A)** Co-immunoprecipitation assays showed that PCAF was bound with histone H4 directly; **(B)** Forced expression of PCAF increased acetylation of histone H4 and AKT phosphorylation in both Huh7 and HepG2 cells, and knockdown of PCAF in Hep3B cells deacetylated histone H4 and activated AKT signaling.

To figure out the underlying mechanism by which PCAF induces cell apoptosis and growth arrest in HCC cells, we tested the effect of PCAF on nuclear acetylation of histone H4 and activation of AKT signaling. As shown in Figure 4A, the co-immunoprecipitation measurement showed that PCAF protein was bound with histone H4 protein directly in Huh7 cells. And the further immunoblotting assay (Figure 4B) found that the level of acetylated histone H4 protein in wild type Hep3B cells with more PCAF expression seemed to be higher than one in Huh7 cells. As expected, Huh7 PCAF cells had higher level of acetylated histone H4 protein than Huh7 Control cells. Consequently, forced expression of PCAF attenuated the phosphorylation of AKT in Huh7 cells. In contrary, knockdown of PCAF reduced the level of acetylation of histone H4 protein in Hep3B cells and increased the phosphorylation of AKT.

To confirm this signaling cascade further, we conducted western immunoblotting assay in HepG2 cells and found that ectopic expression of PCAF in HepG2 cells enhanced acetylation of histon H4 protein and eliminated phosphorylation of AKT protein (Figure 4B).

### PCAF inhibited tumor growth in HCC xenograft model

Based on the above-mentioned evidences showing that PCAF promotes cell apoptosis and growth arrest *in vitro*, we hypothesized that PCAF may impact HCC tumorigenesis *in vivo*. To test this hypothesis, Huh7 PCAF cells and Huh7 Control cells were inoculated subcutaneously into 22 nude mice. Tumor size was measured with calipers every 5 days.

**Figure 5:**
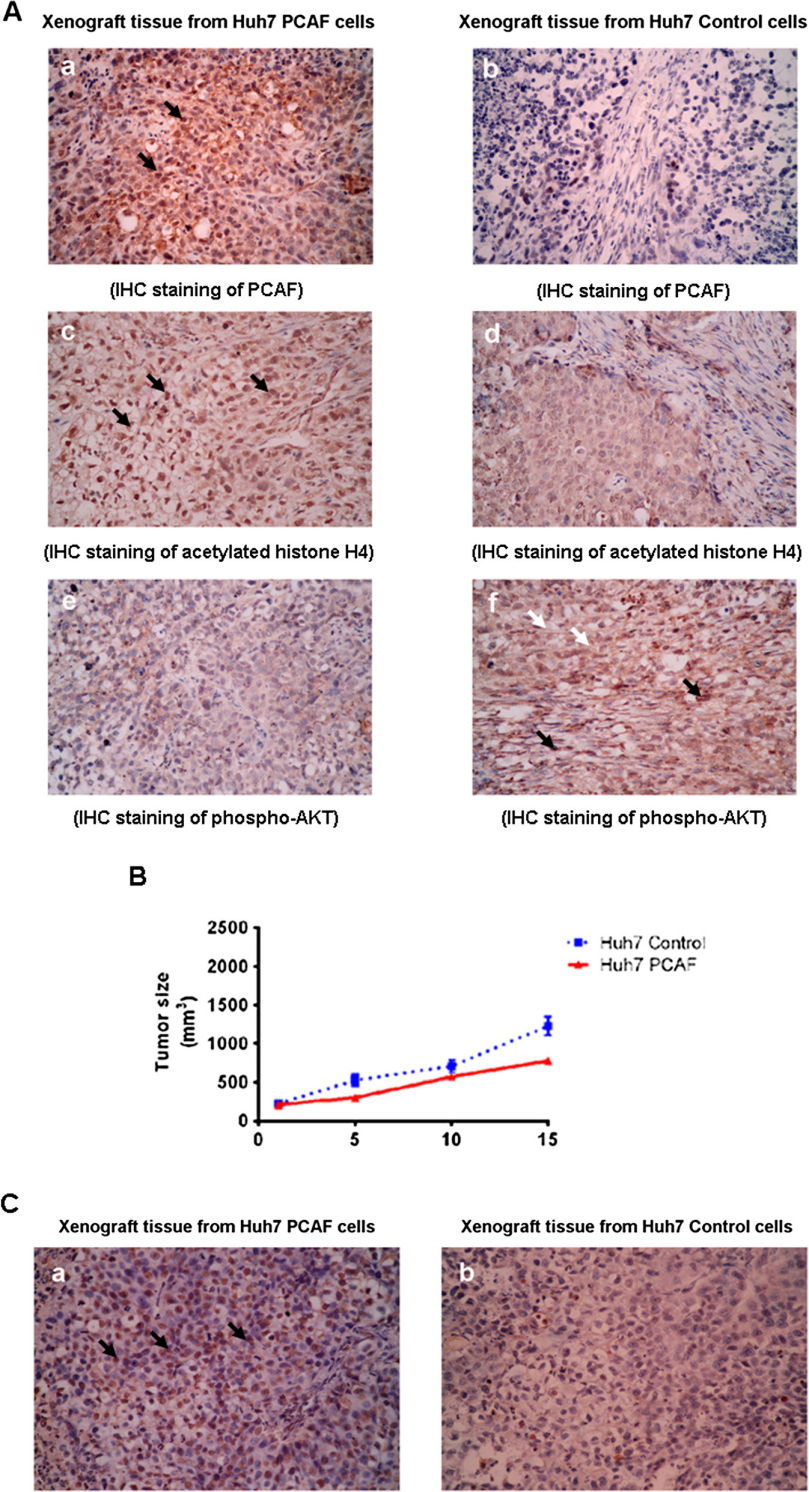
**Forced expression of PCAF inhibited the growth of HCC xenografts, up-regulated histone H4 acetylation, suppressed phosphorylation of AKT, and accelerated cell apoptosis. (A)** IHC experiments was performed to verify the positive expression of PCAF in the xenografts from Huh7 PCAF cells **(a)** and the negative staining of PCAF in the xenografts from Huh7 Control cells **(b)**. The PCAF staining was located in cell nucleus primarily indicated by black arrow. There was more acetylated histone H4 expressing in the xenografts from Huh7 PCAF cells **(c)** than the xenografts from Huh7 PCAF cells **(d)**. The acetylated histone H4 was found mainly in cell nucleus (black arrow). The phosphorylation of AKT protein was upregulated in the xenografts from Huh7 PCAF cells **(e)**, compared to the xenografts from Huh7 Control cells **(f)**. The phospho-AKT expression was detected in both cell nucleus (black arrow) and cytoplasm (white arrow); **(B)** Xenografts from Huh7 PCAF cells grew significantly slower than xenografts from Huh7 Control cells; **(C)** There were more apoptotic cells in the xenograft tissues from Huh7 PCAF cells **(a)** than the xenograft tissues from Huh7 Control cells **(b)**.

As expected, subcutaneous xenografts were detectable in both groups after about 20 days. When the tumor size reached 1000 mm^3^, the mice were sacrificed and the xenografts were harvested. To verify the overexpression of PCAF in the xenografts from Huh7 PCAF cells, immunohistochemistry staining was carried out using the primary antibody against PCAF. As shown in Figure 5A, strong expression of PCAF protein was observed in the xenografts from Huh7 PCAF cells, while negative PCAF staining was found in the xenografts from Huh7 Control cells. To evaluate the signaling cascade found in *in vitro* experiments, we detected the protein expression of acetyl-histone H4 and phospho-AKT in the xenograft tissues. The results showed that there were higher expression of acetyl-histone H4 and lower phospho-AKT expression in the xenograft tissues established from Huh7 PCAF cells (Figure 5A).

Xenografts expressing PCAF grew significantly slower than xenografts from Huh7 Control cells (Figure 5B). Additionally, the TUNEL assay revealed that there were more apoptosis cells in the xenograft tissues from Huh7 PCAF cells (Figure 5C).

## Discussion

Besides the classical genetic mutations (such as chromosomal deletion, chromosomal rearrangements and gene amplifications) have been established to be involved in hepatocarcinogenesis, recent biochemistry researchers have identified that several epigenetic alterations affect the transcription and/or expression of oncogenic proteins and repressors which play important roles in development and progression of HCC [23]. Among them, acetylation of histones emerges as the major epigenetic alteration and contributed fundamentally to transcriptional regulation [24,25] via allowing interconversion between permissive and repressive chromatin structures and domains.

The acetylation level of histones depends on the balance between the activities of HATs and histone deacetylases (HDACs) which are ability to add or remove acetyl groups from the lysines of histones respectively. There are several lines of studies showing that dysregulation of HDACs exists in various cancers including HCC and is responsible for inappropriate transcriptional activation attributed to the progression of these cancers [26,27]. However, there is limited investigation reported about the role of HATs on the pathogenesis of HCC. As aforementioned, PCAF is known as a kind of histone acetyltransferases (HAT), which modulates concurrently multiple cell pathways via acetylating histones and non-histone proteins. In our previous study, PCAF was down-regulated frequently in HCC tissues compared to adjacent liver tissues and associated positively with better survival after liver resection [7]. And it’s down-regulation in HCC tissues was found to be significantly correlated with tumor TNM staging and intrahepatic metastasis. It seems that PCAF functions as a tumor repressor in HCC, which is consistent with its anti-tumor effect in other cancers [28]. In this study, we measured the expression of PCAF in 5 kinds of HCC cell lines and found PCAF expression was relative low in 4 kinds of HCC cells (HepG2, Huh7, PLC/PRF/5, and SKHep1), which indicates that there is limited expression of PCAF in most of HCC cell lines and is consistent with our previous results.

**Figure 6:**
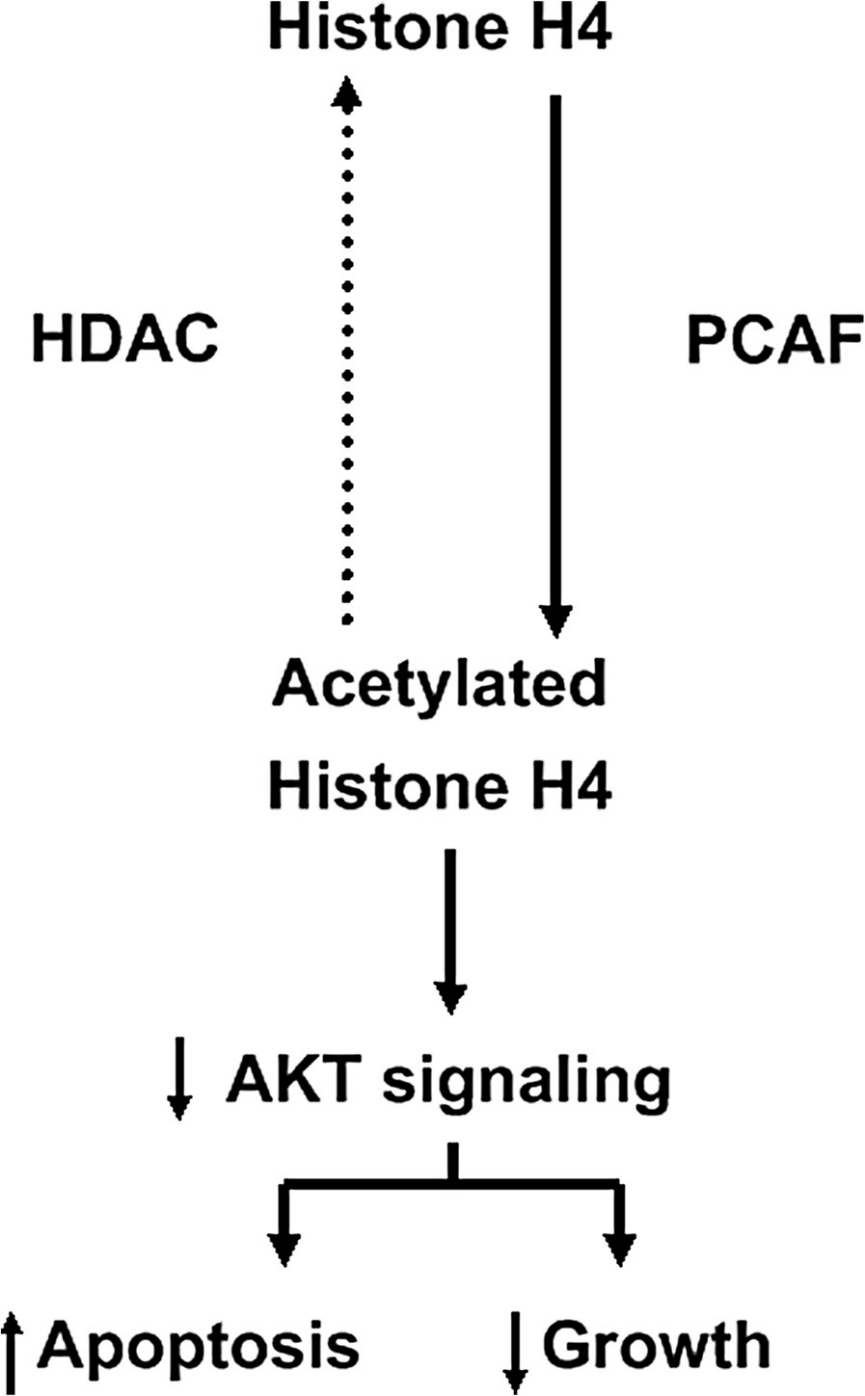
**Working model for the anti-HCC effect of PCAF and downstream pathway.** PCAF promotes apoptosis and inhibits growth of HCC through acetylating histone H4 and inactivating AKT signaling.

PCAF has been found to acetylate histone H4 at lysine 8 [29]. Strikingly, Lai et al. have verified that acetylation of histone H4 induces cell apoptosis and growth arrest via inhibiting AKT signaling [14]. Hence, we tested here the action of PCAF on apoptosis and growth of HCC cells by different ways and found that forced expression of PCAF promoted cell apoptosis and suppressed proliferation of HCC cells. In contrary, knockdown of PCAF repressed cell apoptosis and accelerated HCC cell proliferation. These results supported strongly that PCAF has the anti-HCC function via inducing cell apoptosis and inhibiting cell proliferation. To further figure out the underlying molecular mechanism, we examined the regulatory function of PCAF on AKT signaling. Several studies have shown that AKT signaling is aberrantly hyperactivated in HCC by distinct ways including down-regulation of PIK3IP1 (phosphatidylinositol-3-kinase interacting protein I) [13] and overexpression of COX2 [30]. AKT signaling has been considered to contribute to inhibit cell apoptosis and facilitate cell proliferation. In this study, we found that overexpression of PCAF increased the acetylation level of histone H4 directly and attenuated the phosphorylation of AKT protein. The opposite results were obtained after silencing PCAF in HCC cells. As shown in Figure 6, these data demonstrate that PCAF could play its anti-HCC action through acetylating histone H4 and in turn inactivating AKT signaling, which is also consistent with the conclusion from Lai group that acetylation of histone H4 inhibits AKT signaling and promotes apoptosis in HCC.

In the previous article [31], PCAF was found to induce cell cycle arrest in a P53-dependent manner. In this study, wild type Hep3B cell is a kind of P53-null cell [32] and Huh7 cells [33] expresses mutant p53. Therefore, the pro-apoptotic function of PCAF on these two kinds of cell lines found in this investigation is speculated to be in a p53-independent manner. However, PCAF was also confirmed to rescue DNA-binding and growth-suppressive activity of mutant forms of p53 in various cancers [34]. This mechanism could be involved in the pro-apoptotic action of PCAF in Huh7 cells, which needs be investigated more. Finally, we performed the HCC xenograft experiments and showed that xenograft with high PCAF expression grew slower than one with low PCAF expression significantly, which further proves the anti-HCC effect of PCAF. In addition, the IHC assay in the xenograft tissues verified *in vivo* that PCAF increased acetylation of histone H4 and repressed AKT phosphorylation in HCC cells. The pro-apoptotic effect of PCAF in HCC cells was finally established by TUNEL assay in xenograft tissues.

## Conclusions

In summary, this study shows that PCAF is expressed at the low level in most of HCC cell lines and represses the HCC growth via inducing apoptosis and promoting proliferation. Furthermore, we figure out that PCAF, as a kind of HATs, acetylates histone H4 and inactivates AKT signaling, which could be the underlying molecular mechanism of the pro-apoptotic function of PCAF in HCC. It is well-known that acetylation of histones plays an important role in the pathogenesis of HCC and is determined by the equilibrium between the activities of HATs and HDACs. While some studies have revealed the role of HDACs in HCC, there is limited literature addressing the effect of HATs on the progression of HCC. This study reveals the anti-HCC function of PCAF first and supplies with a new sight to the epigenetic regulation of HCC. PCAF could potentially serve as a clinical biomarker and therapy target for HCC.

## Abbreviations

PCAF: P300/CBP-associated factor; HCC: Hepatocellular carcinoma; PBS: Phosphate buffered saline; HAT: Histone acetyltransferases; FBS: Fetal calf serum; DAPI: 4,6-diamidino-2-phenylindole; qRT-PCR: Quantitative real-time reverse transcription polymerase chain reaction; IHC: Immunohistochemistry; HDACs: Histone deacetylases; PIK3IP1: Phosphatidylinositol-3-kinase interacting protein I; TUNE: Terminal deoxynucleotidyl transferase-mediated dUTP-biotin nick end labeling assay.

## Competing interest

The authors declare that they have no competing interest.

## Authors’ contributions

XZ, WZ, XHG FHD, ZTL and KST carried out the molecular genetic studies, participated in the sequence alignment and drafted the manuscript. YMY and QGL participated in the design of the study and performed the statistical analysis. XZ and XHG conceived of the study, and participated in its design and coordination and helped to draft the manuscript. All authors read and approved the final manuscript.

## Supplementary Material

Additional file 1: Figure S1 (A) The expression of PCAF in HepG2 cells was increased significantly by PCAF expressing plasmid at the level of both mRNA and protein; (B) The percentage of apoptotic cells was increased by more than two-fold after forced expression of PCAF in HepG2 cells; (C) The caspase3/7 activity of HepG2 cells was enhanced dramatically after ectopic expression of PCAF. Click here for file

## References

[B1] Roberts LR Gores GJ Hepatocellular carcinoma: molecular pathways and new therapeutic targets Semin Liver Dis 2005 25 212 225 10.1055/s-2005-871200 15918149

[B2] Parkin DM Bray FI Devesa SS Cancer burden in the year 2000. The global picture Eur J Cancer 2001 37 Suppl 8 S4 S66 11602373 10.1016/s0959-8049(01)00267-2

[B3] El-Serag HB Davila JA Petersen NJ McGlynn KA The continuing increase in the incidence of hepatocellular carcinoma in the United States: an update Ann Intern Med 2003 139 817 823 10.7326/0003-4819-139-10-200311180-00009 14623619

[B4] Yang XJ Ogryzko VV Nishikawa J Howard BH Nakatani Y A p300/CBP-associated factor that competes with the adenoviral oncoprotein E1A Nature 1996 382 319 324 10.1038/382319a0 8684459

[B5] Grunstein M Histone acetylation in chromatin structure and transcription Nature 1997 389 349 352 10.1038/38664 9311776

[B6] Marcus GA Silverman N Berger SL Horiuchi J Guarente L Functional similarity and physical association between GCN5 and ADA2: putative transcriptional adaptors EMBO J 1994 13 4807 4815 7957049 10.1002/j.1460-2075.1994.tb06806.x PMC395419

[B7] Tuo H Zheng X Tu K Zhou Z Yao Y Liu Q Expression of PCAF in hepatocellular carcinoma and its clincial significance Xi Bao Yu Fen Zi Mian Yi Xue Za Zhi 2013 29 297 300 23643089

[B8] MacDonald VE Howe LJ Histone acetylation: where to go and how to get there Epigenetics 2009 4 139 143 10.4161/epi.4.3.8484 19430203

[B9] Timmermann S Lehrmann H Polesskaya A Harel-Bellan A Histone acetylation and disease Cell Mol Life Sci 2001 58 728 736 10.1007/PL00000896 11437234 PMC11337357

[B10] Sun WJ Zhou X Zheng JH Lu MD Nie JY Yang XJ Zheng ZQ Histone acetyltransferases and deacetylases: molecular and clinical implications to gastrointestinal carcinogenesis Acta Biochim Biophys Sin (Shanghai) 2012 44 80 91 10.1093/abbs/gmr113 22194016

[B11] Yasui W Oue N Ono S Mitani Y Ito R Nakayama H Histone acetylation and gastrointestinal carcinogenesis Ann N Y Acad Sci 2003 983 220 231 10.1111/j.1749-6632.2003.tb05977.x 12724227

[B12] Yamashita Y Shimada M Harimoto N Rikimaru T Shirabe K Tanaka S Sugimachi K Histone deacetylase inhibitor trichostatin a induces cell-cycle arrest/apoptosis and hepatocyte differentiation in human hepatoma cells Int J Cancer 2003 103 572 576 10.1002/ijc.10699 12494463

[B13] Carlisi D Vassallo B Lauricella M Emanuele S D’Anneo A Di Leonardo E Di Fazio P Vento R Tesoriere G Histone deacetylase inhibitors induce in human hepatoma HepG2 cells acetylation of p53 and histones in correlation with apoptotic effects Int J Oncol 2008 32 177 184 18097557 10.3892/ijo.32.1.177

[B14] Lai JP Yu C Moser CD Aderca I Han T Garvey TD Murphy LM Garrity-Park MM Shridhar V Adjei AA Roberts LR SULF1 inhibits tumor growth and potentiates the effects of histone deacetylase inhibitors in hepatocellular carcinoma Gastroenterol 2006 130 2130 2144 10.1053/j.gastro.2006.02.056 16762634

[B15] Gupta M Ansell SM Novak AJ Kumar S Kaufmann SH Witzig TE Inhibition of histone deacetylase overcomes rapamycin-mediated resistance in diffuse large B-cell lymphoma by inhibiting Akt signaling through mTORC2 Blood 2009 114 2926 2935 10.1182/blood-2009-05-220889 19641186 PMC2756203

[B16] Yu C Friday BB Lai JP McCollum A Atadja P Roberts LR Adjei AA Abrogation of MAPK and Akt signaling by AEE788 synergistically potentiates histone deacetylase inhibitor-induced apoptosis through reactive oxygen species generation Clin Cancer Res 2007 13 1140 1148 10.1158/1078-0432.CCR-06-1751 17317822

[B17] Brunet A Bonni A Zigmond MJ Lin MZ Juo P Hu LS Anderson MJ Arden KC Blenis J Greenberg ME Akt promotes cell survival by phosphorylating and inhibiting a Forkhead transcription factor Cell 1999 96 857 868 10.1016/S0092-8674(00)80595-4 10102273

[B18] Diehl JA Cheng M Roussel MF Sherr CJ Glycogen synthase kinase-3beta regulates cyclin D1 proteolysis and subcellular localization Genes Dev 1998 12 3499 3511 10.1101/gad.12.22.3499 9832503 PMC317244

[B19] Engelman JA Luo J Cantley LC The evolution of phosphatidylinositol 3-kinases as regulators of growth and metabolism Nat Rev Genet 2006 7 606 619 16847462 10.1038/nrg1879

[B20] Zheng X Gai X Han S Moser CD Hu C Shire AM Floyd RA Roberts LR The human sulfatase 2 inhibitor 2,4-disulfonylphenyl-tert-butylnitrone (OKN-007) has an antitumor effect in hepatocellular carcinoma mediated via suppression of TGFB1/SMAD2 and Hedgehog/GLI1 signaling Genes, Chromosomes Cancer 2013 52 225 236 10.1002/gcc.22022 23109092 PMC3889201

[B21] Zheng X Vittar NB Gai X Fernandez-Barrena MG Moser CD Hu C Almada LL McCleary-Wheeler AL Elsawa SF Vrabel AM The transcription factor GLI1 mediates TGFbeta1 driven EMT in hepatocellular carcinoma via a SNAI1-dependent mechanism PLoS One 2012 7 e49581 10.1371/journal.pone.0049581 23185371 PMC3501480

[B22] Zheng X Yao Y Xu Q Tu K Liu Q Evaluation of glioma-associated oncogene 1 expression and its correlation with the expression of sonic hedgehog, E-cadherin and S100a4 in human hepatocellular carcinoma Mol Med Rep 2010 3 965 970 21472341 10.3892/mmr.2010.375

[B23] Kondoh N Wakatsuki T Hada A Shuda M Tanaka K Arai M Yamamoto M Genetic and epigenetic events in human hepatocarcinogenesis Int J Oncol 2001 18 1271 1278 11351262 10.3892/ijo.18.6.1271

[B24] Eberharter A Becker PB Histone acetylation: a switch between repressive and permissive chromatin. Second in review series on chromatin dynamics EMBO Rep 2002 3 224 229 10.1093/embo-reports/kvf053 11882541 PMC1084017

[B25] Verdone L Caserta M Di Mauro E Role of histone acetylation in the control of gene expression Biochem Cell Biol 2005 83 344 353 10.1139/o05-041 15959560

[B26] Coradini D Speranza A Histone deacetylase inhibitors for treatment of hepatocellular carcinoma Acta Pharmacol Sin 2005 26 1025 1033 10.1111/j.1745-7254.2005.00195.x 16115366

[B27] Coradini D Zorzet S Rossin R Scarlata I Pellizzaro C Turrin C Bello M Cantoni S Speranza A Sava G Inhibition of hepatocellular carcinomas *in vitro* and hepatic metastases *in vivo* in mice by the histone deacetylase inhibitor HA-But Clin Cancer Res 2004 10 4822 4830 10.1158/1078-0432.CCR-04-0349 15269158

[B28] Schiltz RL Nakatani Y The PCAF acetylase complex as a potential tumor suppressor Biochim Biophys Acta 2000 1470 M37 M53 10722926 10.1016/s0304-419x(99)00037-2

[B29] Schiltz RL Mizzen CA Vassilev A Cook RG Allis CD Nakatani Y Overlapping but distinct patterns of histone acetylation by the human coactivators p300 and PCAF within nucleosomal substrates J Biol Chem 1999 274 1189 1192 10.1074/jbc.274.3.1189 9880483

[B30] Leng J Han C Demetris AJ Michalopoulos GK Wu T Cyclooxygenase-2 promotes hepatocellular carcinoma cell growth through Akt activation: evidence for Akt inhibition in celecoxib-induced apoptosis Hepatology 2003 38 756 768 12939602 10.1053/jhep.2003.50380

[B31] Love IM Sekaric P Shi D Grossman SR Androphy EJ The histone acetyltransferase PCAF regulates p21 transcription through stress-induced acetylation of histone H3 Cell Cycle 2012 11 2458 2466 10.4161/cc.20864 22713239 PMC3404877

[B32] Chan KT Lung ML Mutant p53 expression enhances drug resistance in a hepatocellular carcinoma cell line Cancer Chemother Pharmacol 2004 53 519 526 10.1007/s00280-004-0767-4 15004724

[B33] Calvisi DF Simile MM Ladu S Frau M Evert M Tomasi ML Demartis MI Daino L Seddaiu MA Brozzetti S Activation of v-Myb avian myeloblastosis viral oncogene homolog-like2 (MYBL2)-LIN9 complex contributes to human hepatocarcinogenesis and identifies a subset of hepatocellular carcinoma with mutant p53 Hepatology 2011 53 1226 1236 10.1002/hep.24174 21480327

[B34] Perez RE Knights CD Sahu G Catania J Kolukula VK Stoler D Graessmann A Ogryzko V Pishvaian M Albanese C Avantaggiati ML Restoration of DNA-binding and growth-suppressive activity of mutant forms of p53 via a PCAF-mediated acetylation pathway J Cell Physiol 2010 225 394 405 10.1002/jcp.22285 20589832 PMC3614009

