# Correction: Histone acetyltransferase PCAF Up-regulated cell apoptosis in hepatocellular carcinoma via acetylating histone H4 and inactivating AKT signaling

**DOI:** 10.1186/s12943-022-01637-2

**Published:** 2022-09-28

**Authors:** Xin Zheng, Xiaohong Gai, Feihu Ding, Zhongtang Lu, Kangsheng Tu, Yingmin Yao, Qingguang Liu

**Affiliations:** Department of Hepatobiliary Surgery, the First Affiliated Hospital of Xi’an Jiaotong University, 277 Yanta West Road, Xi’an, 710061 Shaanxi China

**Correction: Mol Cancer 12, 96 (2013)**

**https://doi.org/10.1186/1476-4598-12-96**

In our BMC Research publication in Molecular Cancer entitled ‘Histone acetyltransferase PCAF Up-regulated cell apoptosis in hepatocellular carcinoma via acetylating histone H4 and inactivating AKT signaling’ [1], we regret the errors in Figure 2D and Figure 3D in the printed version. Specifically, the flow cytometry images in Figure 2D and Figure 3D were inadvertently placed by mistake. We have double-checked the original data and found that the inadvertent errors occurred during picture compilation. The corrected Figures 2 and 3 are given here, and this correction does not change the scientific conclusions of the article.

**Fig. 2:**
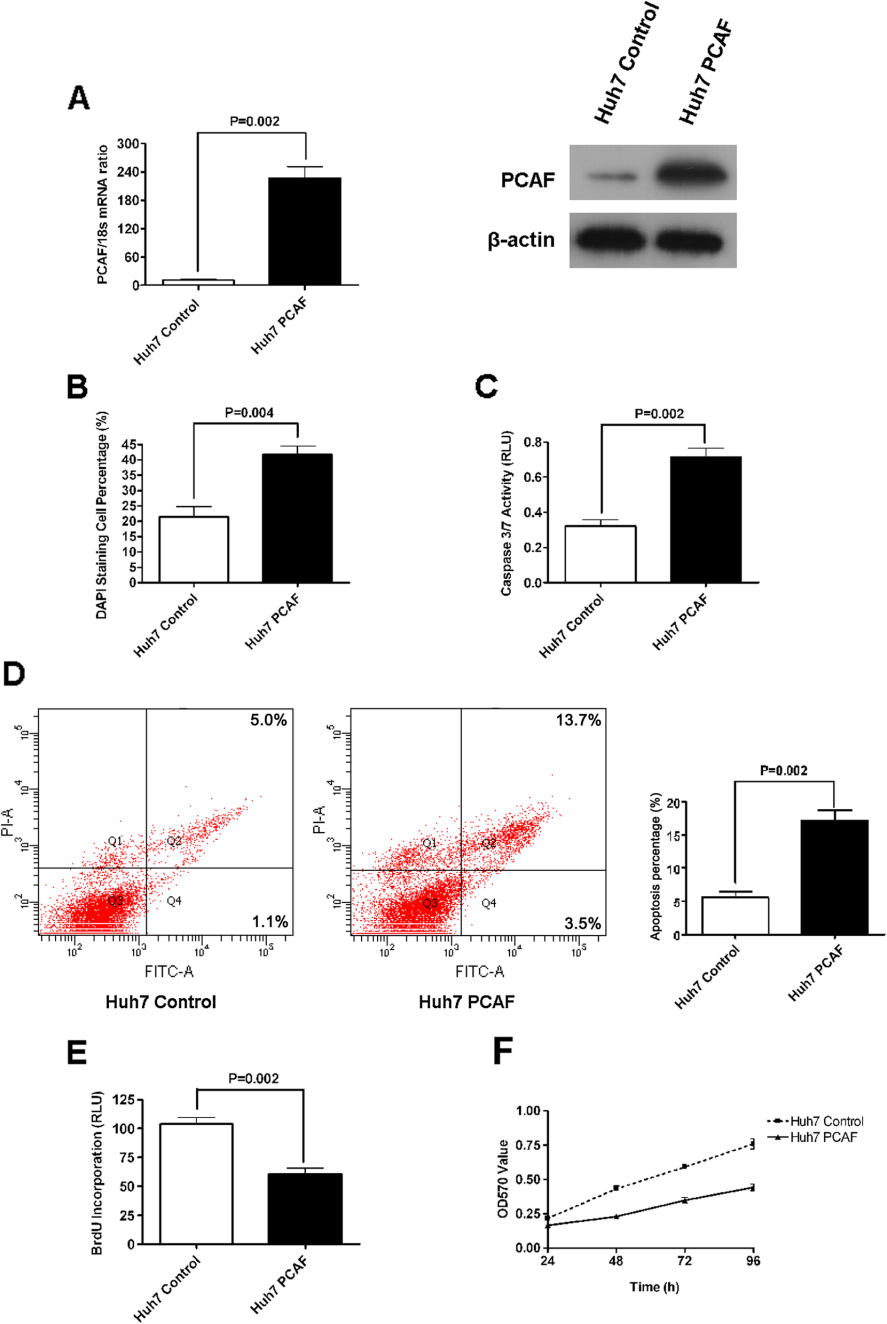
Overexpression of PCAF induced cell apoptosis and repressed proliferation in Huh7 cells. **(A)** At the levels of both mRNA and protein, PCAF expression is increased by PCAF expressing plasmid in Huh7 cells; **(B)** Forced expression of PCAF induced a significant increase in apoptosis of Huh7 cells as assessed by staining with DAPI followed by fluorescence microscopy (*P* < 0.004); **(C)** The activity of the pro-apoptotic caspase 3 and 7 also showed up-regulated after ectopic expression of PCAF (*P* = 0.002); **(D)** Flow cytometry assay showed that the percents of early apoptosis cells and late apoptosis cells was increased 2–3 folds in Huh7 PCAF cells than in Huh7 Control cells (*P* = 0.002); **(E)** Cell proliferation as measured by BrdU incorporation was inhibited by forced expression of PCAF(*P* = 0.002); **(F)** As assessed by MTT assays, forced expression of PCAF was found to reduce viability of Huh7 cells at all four time points significantly

**Fig. 3:**
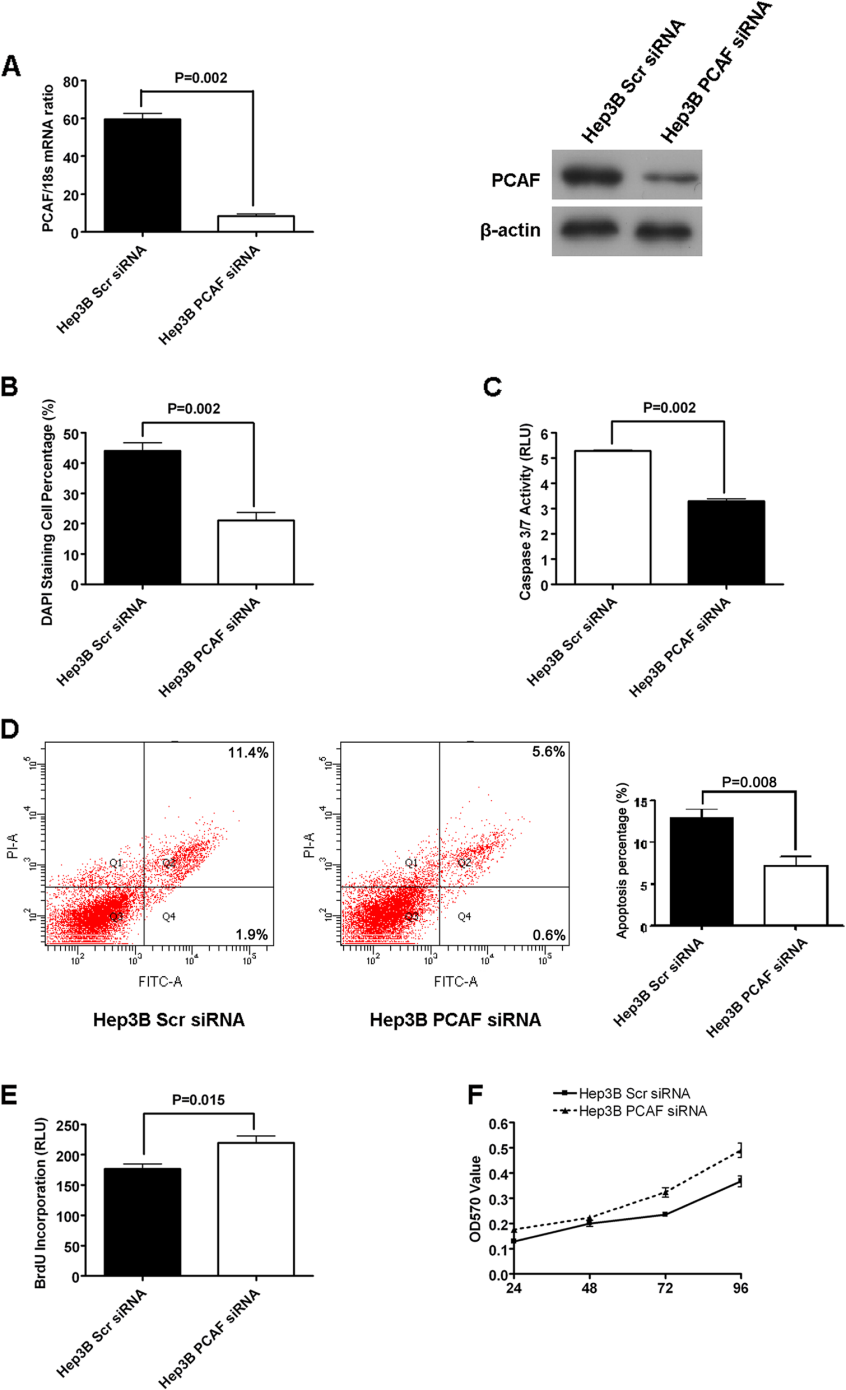
Knockdown of PCAF suppressed cell apoptosis and promoted proliferation in Hep3B cells. **(A)** The mRNA and protein expression of PCAF is down-regulated by siRNA structure against PCAF in Hep3B cells; **(B)** DAPI staining assay showed that knockdown of PCAF repressed cell apoptosis of Hep3B cells significantly (*P* = 0.002); **(C)** The activity of caspase 3/7 was decreased greatly by knockdown of PCAF in Hep3B cells (*P* = 0.002); **(D)** The percents of both early apoptotic cells and late apoptotic cells of Hep3B PCAF siRNA cells was decreased by around 50% as assessed by flow cytometry (*P* = 0.008); **(E)** Silencing PCAF was found to promote proliferation of Hep3B cells in BrdU incorporation ELISA assay apparently; **(F)** MTT assay displayed that knockdown of PCAF facilitated cell growth of Hep3B cells significantly

The authors all agree to this Corrigendum and are grateful to the Editor of Molecular Cancer for allowing them to have the opportunity to correct these errors. The authors would like to apologize for any inconvenience caused.
