# Supplementary figures and images for: Histone acetyltransferase PCAF Up-regulated cell apoptosis in hepatocellular carcinoma via acetylating histone H4 and inactivating AKT signaling
Source: Mol Cancer. 2013 Aug 27;12:96. doi: 10.1186/1476-4598-12-96 (PMC3847488; doi:10.1186/1476-4598-12-96)

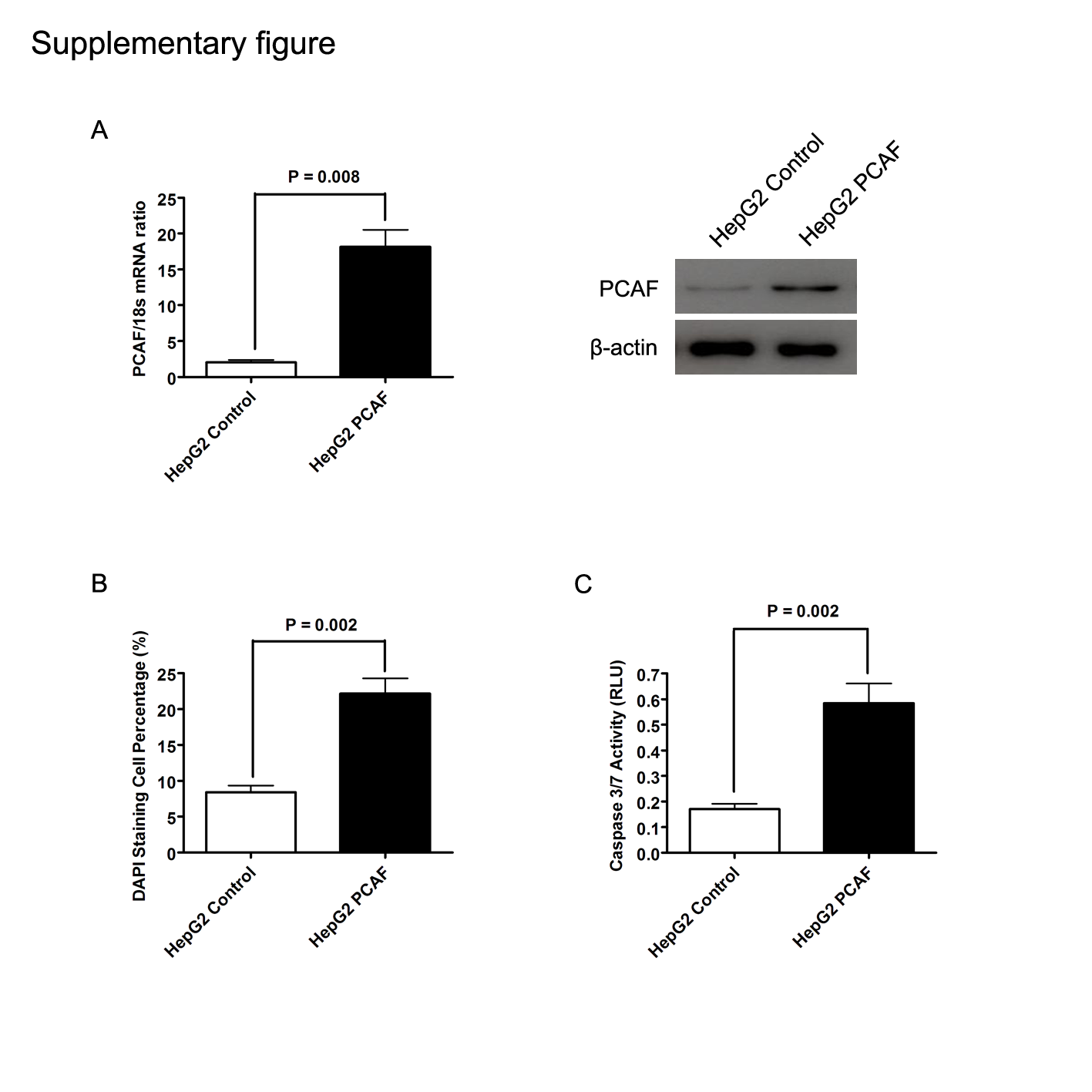

Supplement: Additional file 1: Figure S1 — (A) The expression of PCAF in HepG2 cells was increased significantly by PCAF expressing plasmid at the level of both mRNA and protein; (B) The percentage of apoptotic cells was increased by more than two-fold after forced expression of PCAF in HepG2 cells; (C) The caspase3/7 activity of HepG2 cells was enhanced dramatically after ectopic expression of PCAF. [file 1476-4598-12-96-S1.tiff]
